# Age-Dependent Switch of the Role of Serotonergic 5-HT_1A_ Receptors in Gating Long-Term Potentiation in Rat Visual Cortex* In Vivo*

**DOI:** 10.1155/2016/6404082

**Published:** 2016-05-10

**Authors:** Peter J. Gagolewicz, Hans C. Dringenberg

**Affiliations:** ^1^Center for Neuroscience Studies, Queen's University, Kingston, ON, Canada K7L 3N6; ^2^Department of Psychology, Queen's University, Kingston, ON, Canada K7L 3N6

## Abstract

The rodent primary visual cortex (V1) is densely innervated by serotonergic axons and previous* in vitro *work has shown that serotonin (5-HT) can modulate plasticity (e.g., long-term potentiation (LTP)) at V1 synapses. However, little work has examined the effects of 5-HT on LTP under* in vivo *conditions. We examined the role of 5-HT on LTP in V1 elicited by theta burst stimulation (TBS) of the lateral geniculate nucleus in urethane-anesthetized (adult and juvenile) rats. Thalamic TBS consistently induced potentiation of field postsynaptic potentials (fPSPs) recorded in V1. While 5-HT application (0.1–10 mM) itself did not alter LTP levels, the broad-acting 5-HT receptor antagonists methiothepin (1 mM) resulted in a clear facilitation of LTP in adult animals, an effect that was mimicked by the selective 5-HT_1A_ receptor antagonist WAY 100635 (1 mM). Interestingly, in juvenile rats, WAY 100635 application inhibited LTP, indicative of an age-dependent switch in the role of 5-HT_1A_ receptors in gating V1 plasticity. Analyses of spontaneous electrocorticographic (ECoG) activity in V1 indicated that the antagonist-induced LTP enhancement was not related to systematic changes in oscillatory activity in V1. Together, these data suggest a facilitating role of 5-HT_1A_ receptor activation on LTP in the juvenile V1, which switches to a tonic, inhibitory influence in adulthood.

## 1. Introduction

Long-term potentiation (LTP), a form of brain plasticity characterized by a long-lasting increase in synaptic coupling of neurons, has been suggested as a candidate mechanism mediating processes of learning and memory in the nervous system [1, 2]. First characterized in the hippocampal formation [3], LTP has now been shown to occur at synapses throughout the nervous system, including cortical sensory areas such as the primary visual (V1), auditory, and somatosensory cortices [4–6]. In V1, LTP has been successfully induced under both* in vitro *and* in vivo *conditions, with work in slice preparations indicating that LTP is limited to a well-defined time window during early postnatal life, after which V1 synapses become resistant to LTP induction [7–12]. Interestingly, under* in vivo *conditions, LTP is readily induced in the fully matured V1 of adult rodents [13–15], indicative of some fundamental differences in the induction of LTP in V1 between* in vivo *and* in vitro *conditions.

An important aspect of LTP regulation lies in the role of various neuromodulators present in the extracellular environment. For example, acetylcholine (ACh) exerts a powerful, modulatory effect by enhancing LTP in V1, an effect that is apparent under both* in vitro* and* in vivo* conditions and for a number of different (e.g., weak and strong) LTP induction protocols [14, 16, 17]. A similar, facilitating effect on LTP is also seen with histamine application directly in V1 of rats* in vivo *[18], highlighting the importance of a variety of neuromodulators as gating mechanism for the induction of plasticity at cortical synapses [19–21]. The central serotonergic (5-hydroxytryptamine, 5-HT) system has also been implicated in the modulation of cortical synaptic plasticity. Serotonergic fibers originating in the dorsal and median raphe nuclei provide a dense innervation of the entire forebrain, including V1 [22–25]. The presence of 5-HT receptors in V1 has been confirmed by radioligand-binding experiments [26, 27] and* in situ* hybridization techniques [28], implying functionality of the 5-HT input to V1.

Previous work on the role of 5-HT in the regulation of LTP in V1 has yielded inconsistent results. In V1 slices obtained from kittens (40–80 days old), 5-HT application facilitated LTP induction in layer 4 neurons, effects that were absent in older (more than 120 days) animals [8, 9]. Similarly, in the immature rat V1* in vitro*, LTP of layer 2/3 neurons elicited by layer 4 stimulation was impaired by 5-HT depletion or bath application of antagonists of 5-HT_1A_ or 5-HT_2_ receptors [29]. Together, these observations suggest a facilitating role for 5-HT in LTP induction in the immature V1, findings that have recently been extended to more mature (8–10 weeks old) rats [12].

Surprisingly, others have reported an inhibition of LTP in layers 2/3 of V1 slices obtained from juvenile rats following bath application of 5-HT [12, 30]. These results have led to the suggestion that the developmentally increasing serotonergic tone in V1 is responsible for the loss of LTP throughout cortical maturation [10, 31], at least under* in vitro *conditions (see above). However, it is unclear how to reconcile this assumption with data demonstrating serotonergic facilitation of LTP in the more mature V1 [12], as well as observations that both LTP and ocular dominance plasticity in V1 can be reinstated in adult rodents following treatment with the selective serotonin reuptake inhibitor (SSRI) fluoxetine [11].

The above summary of prior work suggests that the role of 5-HT in the regulation of LTP in V1 is not fully understood and may also depend on the age and developmental status of the animal. Further, there is a clear lack of information regarding serotonergic effects on LTP assessed in the intact brain* in vivo*, which appears important, given the differences in LTP induction in the mature V1 between* in vivo *and* in vitro *preparations (see above). To clarify some of these unresolved issues, we examined the role of 5-HT and some 5-HT receptors in the induction of LTP in the V1 of juvenile and adult rats using intact, urethane-anesthetized animals, an experimental preparation that continues to express LTP well into adulthood [13–15].

## 2. Materials and Methods

### 2.1. Subjects

All procedures adhered to the guidelines of the Canadian Council on Animal Care and were approved by the Queen's University Animal Care Committee. Experiments were conducted on adult (300–550 g; >70 days old) or juvenile (190–240 g; mean age: 45 days, age range: 42–48 days) male Long-Evans rats (Charles River Laboratories, Saint-Constant, QC, Canada). The animals were housed in a colony room under a reversed 12 : 12-h light cycle (lights on at 19:00 h), with water and food access* ad libitum*. Each animal was used for only one experiment. All efforts were made in order to minimize animal suffering and the number of animals employed for these experiments.

### 2.2. Surgery

Each animal was deeply anesthetized with 2.0 g/kg urethane (Sigma-Aldrich, Oakville, ON, Canada), administered intraperitoneally (i.p.) as four doses of 0.5 g/kg each, given every 20 min. Additional 0.5 g/kg supplements of urethane were administered when necessary. Fifteen minutes prior to the start of surgery, the local analgesic bupivacaine (Marcaine; Hospira Healthcare Corporation, Montreal, QC, Canada) was administered subcutaneously to the skin and tissue along the incision line over the skull (two or three injections; total of 5 mg/kg). Throughout the surgical procedure and experiment, body temperature was monitored with a rectal thermometer and maintained at 37 ± 1°C by means of an electrical heating pad and fleece insulating blankets surrounding the body.

After anesthesia induction, a rat was placed in a stereotaxic apparatus, the skull bone was exposed, and small holes were drilled overlying the following areas (all measurements taken from bregma and the skull surface): lateral geniculate nucleus (LGN), anterior-posterior −4.1 mm, lateral +4.1 mm, and ventral −4.8 to −5.1 mm; V1, anterior-posterior −7.6 mm, lateral +3.6 mm, and ventral −0.8 to −1.2 mm. Two additional holes were drilled in the bone overlying the left and right cerebellum to secure jewelry screws, which served as ground and reference connections. A concentric, bipolar stimulation electrode (SNE-100; Rhodes Medical Instruments, David Kopf Instruments, Tujunga, CA) was lowered into the LGN, while a monopolar recording electrode (125 *μ*m diameter Teflon-insulated stainless steel wire) was placed in the superficial layers of V1. The final, ventral depth of both electrodes was adjusted to yield maximal amplitude field postsynaptic potentials (fPSPs) recorded in V1 in response to single-pulse LGN stimulation.

### 2.3. Electrophysiology

Stimulation of the LGN (single 0.2 ms pulses) was achieved by connecting the stimulation electrode to a stimulus isolation unit (ML 180 Stimulus Isolator; AD Instruments, Toronto, ON, Canada) providing a constant current output. The fPSPs in V1 were recorded differentially, with the recording electrode referenced against a screw in the bone overlying the cerebellum. The V1 signal was amplified (half-amplitude filters at 0.3 Hz to 1 kHz), digitized (10 kHz) by an A/D converter (PowerLab 4/s system running Scope software v. 3.6.5; AD Instruments), and stored for offline analysis.

**Figure 1:**
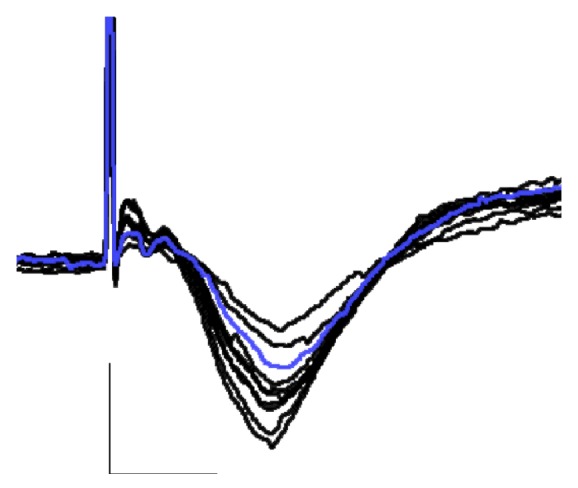
Typical fPSPs recorded in V1 in response to single-pulse stimulation of the ipsilateral LGN in a urethane-anesthetize rat. fPSPs were recorded during input-output stimulation (0.1 to 1.0 mA stimulation current); note the increase in fPSP amplitude with increasing stimulation intensities. The blue trace (elicited by 0.3 mA) was the intensity used for the subsequent data collection (bottom; calibration bars indicate 0.5 mV vertical and 10 ms horizontal).

For each rat, an input-output curve was established by stimulating the LGN at increasing intensities (0.1–1.0 mA in 0.1 mA increments) and the intensity yielding approximately 50–60% of the maximal fPSP amplitude was then used for the remainder of the experiment (see Figure 1).

Cortical fPSPs were recorded every 30 s until 30 min of stable baseline recordings were achieved (≤5% difference between successive data points for fPSPs averaged over 10 min epochs). Subsequently, theta burst stimulation (TBS) was delivered to the LGN, consisting of five single pulses (at 100 Hz) per burst, with bursts repeated at 5 Hz for a total of 10 bursts (pulse intensity and duration were the same as stated above). Recordings of fPSPs (every 30 sec) continued for 2 h following TBS delivery.

In all experiments, spontaneous electrocorticographic (ECoG) activity was also recorded through the same V1 electrode used for fPSP recordings. For the ECoG, the cortical signal was digitized (200 Hz), band-pass filtered (0.3–50 Hz), and analyzed offline for peak power in the main frequency bands (low delta, 0.5–1 Hz; delta, 1–4 Hz; theta, 4–8 Hz; alpha, 8–12 Hz; beta, 12–20 Hz; and gamma, 20–40 Hz). The ECoG was sampled (5 sec epochs) prior to the onset of fPSP baseline recordings and at the end of experiment, that is, 120 min after TBS.

### 2.4. Pharmacology

To investigate the roles of 5-HT and different 5-HT receptors, independent groups of animals received one of the following drug treatments: 5-hydroxytryptamine hydrochloride (5-HT; 0.1 or 10 mM; Sigma-Aldrich);* N*-[2-[4-(2-methoxyphenyl)-1-piperazinyl]ethyl]-*N*-2-pyridinylcyclohexanecarboxamide maleate (WAY 100635; 1 mM; Tocris Bioscience, Ellisville, MO, USA); 1-[10,11-dihydro-8-(methylthio) dibenzo(*Z*)[*b*, *f*]thiepin-10-yl]-4-methylpiperazine maleate (methiothepin; 1 mM; Tocris Bioscience); 8-hydroxy-2-(di-n-propylamino)-tetralin hydrobromide (8-OH-DPAT; 1 mM; Tocris Bioscience). WAY 100635 and 8-OH-DPAT act as potent 5-HT_1A_ receptor antagonist and agonist, respectively [32–34], and 8-OH-DPAT also exhibits an affinity for 5-HT_7_ receptors (see [35]). Methiothepin is a potent 5-HT_2_ receptor antagonist but also acts as an antagonist at 5-HT_1_, 5-HT_5A_, 5-HT_5B_, 5-HT_6_, and 5-HT_7_ receptors [36, 37]. All compounds were dissolved in artificial cerebrospinal fluid (aCSF), consisting of 118.3 mM NaCl, 4.4 mM KCl, 1.2 mM MgSO_4_, 1.0 mM NaH_2_PO_4_, 2.5 mM CaCl_2_, 22.1 mM NaHCO_3_, and 10.0 mM glucose, with the exception of methiothepin, which was dissolved in either a mixture of aCSF and dimethyl sulfoxide (DMSO; *n* = 5), saline (*n* = 4) or aCSF and distilled water (*n* = 4; there were no significant differences in LTP among these different vehicle solutions and rats were combined into a single methiothepin group).

Drugs were applied locally in V1 by means of reverse microdialysis. The dialysis probe (Mab 6.14.2, 15,000-Da cut-off polyether sulfone membrane, outer diameter 0.6 mm; S.P.E. Limited, North York, ON, Canada) was mounted immediately adjacent to the V1 recording electrode, with the probe tip extending approximately 1 mm past the electrode tip. Drug concentrations reaching the brain are estimated to be ~10% of the aCSF content within the vicinity of the probe membrane (about 1 mm; [38, 39]). The dialysis probe was connected to a 2.5mL Hamilton syringe using FEP microtubing (S.P.E. Limited). The syringe was driven by a microdialysis pump (CMA 402; CMA Microdialysis, Solna, Sweden) at a flow rate of 1 *μ*L/min, with perfusion beginning 20 min prior to the acquisition of baseline fPSP recordings and continuing throughout the entire experiment.

### 2.5. Histology

At the conclusion of electrophysiological data acquisition, all animals received a supplementary dose (1.0 mL) of urethane and, after 5–10 min, were perfused through the heart with 0.9% saline (~50 mL) followed by 10% formalin (~100 mL). The brains were removed and stored in 10% formalin for a minimum of 24 h before sectioning (40 *μ*m slices) with a cryostat. Slices were then mounted onto microscope slides and inspected with a digital microscope to verify electrode placements. Histological inspections and decisions on the accuracy of electrode placements were made by an experimenter who was blind to the results of individual animals. Data from inaccurate placements were omitted from this study (77 and 39 rats included and rejected due to missed placements, resp.).

### 2.6. Data Analysis

Cortical fPSPs were analyzed with Scope software (v. 3.6.5; AD Instruments). With the electrode configuration employed in the present study, fPSPs elicited in V1 consisted of a predominant, large amplitude, negative-going component. The amplitude of this component was automatically detected and computed with Scope software (using the Data Pad function) by measuring the voltage difference between the maximal fPSP negativity and the baseline voltage sampled immediately prior to the stimulation artifact. Once individual fPSPs were analyzed in this manner, they were averaged over successive 10 min intervals (i.e., 20 fPSPs/interval) and then normalized by dividing them by the average baseline (pre-TBS) amplitude of each animal. Amplitude data are presented as mean ± standard error of the mean (SEM) and were analyzed with a repeated-measures analysis of variance (ANOVA) and followed up, where statistically appropriate, with Bonferroni* post hoc* tests or unpaired Student's* t*-tests using SPSS software (v. 15.0; SPSS, Colorado Springs, CO, USA). The analysis was conducted with time (10 min averaging epochs) as the repeated/within-subjects factor and drug group as the between-subjects factor.

Five-second epochs of ECoG activity were analyzed using Chart software (v. 5.5.6; AD Instruments). For each epoch, spectral analysis was used to determine the % of total power contained in each of the following frequency bands: 0.5–1 Hz, 1–4 Hz, 4–8 Hz, 8–12 Hz, 12–20 Hz, and 20–40 Hz. Data from individual experiments were averaged across treatment groups and statistically analyzed by ANOVA using SPSS software.

## 3. Results

### 3.1. Characteristics of fPSPs in V1 Elicited by LGN Stimulation

In urethane-anesthetized animals, single-pulse stimulation of the LGN reliably elicited fPSPs in the ipsilateral V1 (Figure 1). In agreement with previous work using this experimental preparation [14, 15, 18], fPSPs were composed mainly of a large amplitude (up to 0.5 mV), negative component, with a latency to peak of 16–18 ms following LGN stimulation (Figure 1). These fPSPs appear to reflect, for the most part, excitatory current sinks originating in layers 2/3 of V1 [13].

### 3.2. Effects of Pharmacological Treatments on Baseline (Pre-TBS) fPSP Amplitude

**Figure 2:**
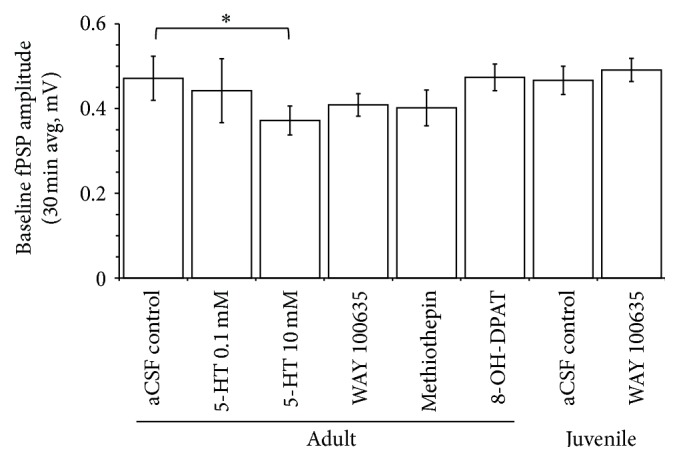
The effect of application (in V1 by means of reverse microdialysis; application started 20 min prior to the onset of recordings) of either aCSF (adult and immature animals), 5-HT (0.1 and 10 mM; adults), WAY 100635 (1 mM; adults and immature), methiothepin (1 mM; adults), or 8-OH-DPAT (1 mM; adults) on baseline (pre-TBS delivery) amplitude of fPSP recorded in V1. Each bar represents averaged fPSP amplitudes during 30 min (i.e., 60 fPSPs) of baseline recordings. The high concentration of 5-HT (10 mM) resulted in a significant (*P* < 0.05; *t*-test) depression of fPSP amplitude compared to aCSF application. None of the other pharmacological treatments resulted in a significant change of fPSP amplitudes relative to aCSF application. ^*∗*^ indicates a significant (*P* < 0.05, *t*-test) difference between the two groups.

Levels of LTP may be influenced by differences in baseline (i.e., prior to LTP induction) synaptic strength. Thus, we initially computed and compared the baseline amplitude of fPSPs in all experimental groups prior to the delivery of TBS to induce LTP. As shown in Figure 2, for all groups, baseline fPSP amplitudes were in the range of 0.37 to 0.47 mV. Application of the high concentration of 5-HT (10 mM) resulted in a modest, but significant (*P* < 0.05, *t*-test) suppression of fPSP amplitude relative to rats receiving aCSF application in V1. None of the other pharmacological treatments produced a significant change in fPSP amplitude relative to the aCSF condition (Figure 2).

### 3.3. Effect of 5-HT on LTP in Adult Rats

**Figure 3:**
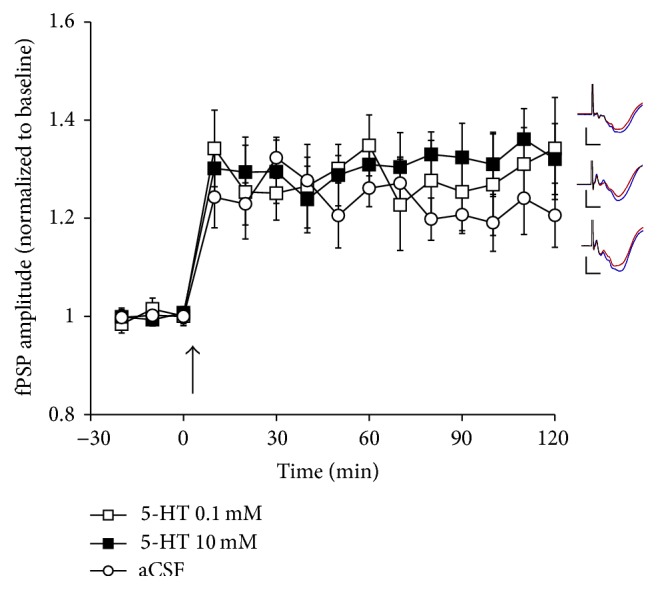
The effect of application (in V1 by means of reverse microdialysis) of either aCSF (*n* = 8) or 5-HT (0.1 or 10 mM; *n* = 5 and 7, resp.) on LTP following TBS (at arrow) of the LGN in urethane-anesthetized rats. Application of 5-HT did not result in any significant changes in LTP relative to rats receiving aCSF. Inserts depict typical fPSPs before (red) and after (blue) LTP induction for animals in the presence of 0.1 mM 5-HT (top), 10 mM 5-HT (middle), or aCSF (bottom; calibration bars indicate 0.5 mV vertical and 10 ms horizontal; each fPSP trace is an average of 30 min of continuous recording).

To determine the effects of 5-HT application on LTP, separate groups of adult animals received application of either aCSF or 5-HT at 0.1 mM or 10 mM. Application occurred locally in V1 by means of reverse microdialysis, with the dialysis probe situated immediately adjacent to the cortical recording electrode. During aCSF application (*n* = 8), TBS of the LGN reliably induced LTP, with fPSP amplitude increasing to 121 ± 4% of baseline (Figure 3; all values reported here are averages of the last 30 min of the experiment, i.e., from 91 to 120 min after TBS delivery). During application of 0.1 mM (*n* = 5) or 10 mM of 5-HT (*n* = 7), fPSP amplitude reached 131 ± 5% and 133 ± 4% of the baseline, respectively (Figure 3). These values appeared higher than those observed during aCSF application, but this difference did not reach statistical significance, as revealed by the ANOVA showing a significant main effect of time, but not of group (*F*_2,17_ = 0.5, *P* > 0.6) or a time × group interaction (*F*_10,88_ = 0.91, *P* > 0.5). Thus, under the present, experimental conditions, 5-HT did not exert a significant influence on LTP induction in the thalamocortical visual system of adult rats.

### 3.4. Effect of 5-HT Receptor Antagonists or a 5-HT_1A_ Agonist on LTP in Adult Rats

**Figure 4:**
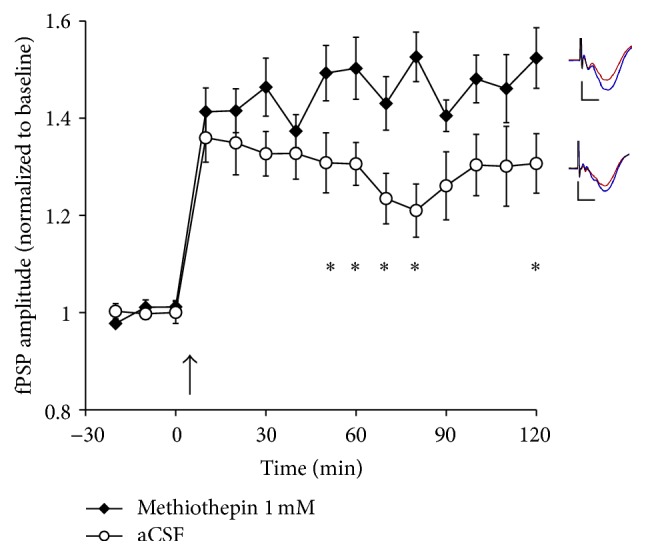
The effect of application (in V1 by means of reverse microdialysis) of either aCSF/vehicle (*n* = 11; see text for detail regarding this control group) or methiothepin (1 mM, *n* = 13) on LTP following TBS (at arrow) of the LGN in urethane-anesthetized rats. Application of methiothepin resulted in a significant increase in LTP relative to control animals. Inserts depict typical fPSPs before (red) and after (blue) LTP induction in the presence of methiothepin (top) and control animals (bottom; calibration bars indicate 0.5 mV vertical and 10 ms horizontal; each fPSP trace is an average of 30 min of continuous recording). ^*∗*^ indicates a significant (*P* < 0.05, *t*-test) difference between the two groups.

To examine a possible effect of 5-HT blockade on LTP, we initially applied the broad-acting 5-HT receptor antagonist methiothepin (1.0 mM) in a separate group of adult animals (*n* = 13). In these rats, TBS of the LGN resulted in an increase of fPSP amplitude to 149 ± 3% of baseline during the last 30 min of the experiment (Figure 4). Given that methiothepin was dissolved in several, different vehicle solutions (see Section 2.4.) a new group of control rats was also examined (*n* = 11; consisting of aCSF/DMSO, *n* = 4; saline, *n* = 3; and aCSF/H_2_0, *n* = 4). In these rats, fPSP amplitude reached 130 ± 4% of baseline during the final 30 min of the experiment (Figure 4). An ANOVA revealed that methiothepin enhanced LTP levels, as indicated by significant main effects of time (*F*_5,116_ = 38.8, *P* < 0.001) and group (*F*_1,22_ = 6.38, *P* < 0.02), as well as a significant time × group interaction (*F*_3,116_ = 3.47, *P* ≤ 0.005).

**Figure 5:**
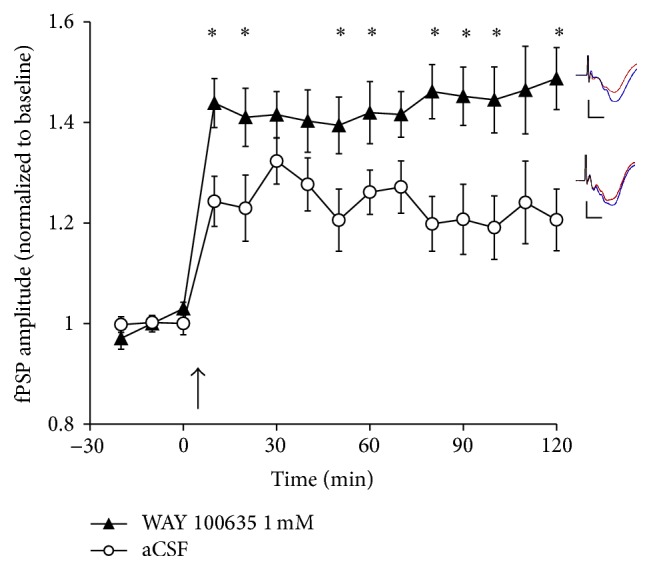
The effect of application (in V1 by means of reverse microdialysis) of either aCSF (*n* = 8; same group as in Figure 3) or WAY 100635 (1 mM, *n* = 10) on LTP following TBS (at arrow) of the LGN in urethane-anesthetized rats. Application of WAY 100635 resulted in a significant increase in LTP relative to control animals. Inserts depict typical fPSPs before (red) and after (blue) LTP induction in the presence of WAY 100635 (top) and aCSF (bottom; calibration bars indicate 0.5 mV vertical and 10 ms horizontal; each fPSP trace is an average of 30 min of continuous recording). ^*∗*^ indicates a significant (*P* < 0.05, *t*-test) difference between the two groups.

In order to further characterize the LTP enhancement seen with 5-HT receptor blockade, we tested the effect of V1 application of WAY 100635 (1 mM, *n* = 10), a highly selective antagonist at 5-HT_1A_ receptors [31, 32]. In this group of rats, TBS increased fPSP amplitude to 146 ± 4% of baseline (Figure 5). This level of LTP was significantly higher than that in rats receiving application of aCSF (the same group as in Figure 3; 121 ± 4% of the baseline, *n* = 8), as indicated by the main effects of time (*F*_5,88_ = 22.9, *P* < 0.001) and group (*F*_1,16_ = 10.51, *P* < 0.005), and by a time × group interaction (*F*_5,88_ = 2.75, *P* ≤ 0.02).

**Figure 6:**
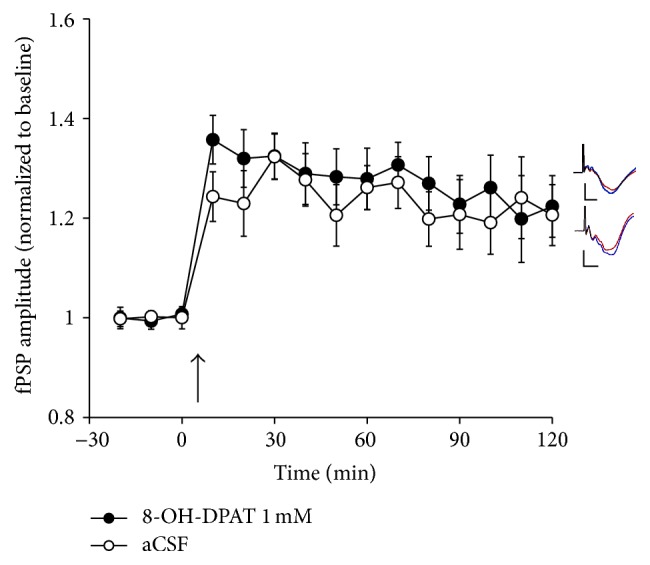
The effect of application (in V1 by means of reverse microdialysis) of either aCSF (*n* = 8; same group as in Figure 3) or 8-OH-DPAT (1 mM, *n* = 8) on LTP following TBS (at arrow) of the LGN in urethane-anesthetized rats. Application of 8-OH-DPAT did not result in a significant change in LTP relative to animals receiving aCSF. Inserts depict typical fPSPs before (red) and after (blue) LTP induction in the presence of 8-OH-DPAT (top) and aCSF (bottom; calibration bars indicate 0.5 mV vertical and 10 ms horizontal; each fPSP trace is an average of 30 min of continuous recording).

Based on the results reported above, it appears that 5-HT_1A_ receptors exert an inhibitory effect over LTP induction in the mature V1. To further examine the role of these receptors, we applied the highly selective 5-HT_1A_ receptor agonist 8-OH-DPAT in V1 (1 mM, *n* = 8). In these animals, TBS elicited LTP, with fPSP amplitude reaching 123 ± 3% of baseline during the last 30 min of the experiment (Figure 6). These values were not significantly different from those in rats receiving aCSF application (same group as in Figure 3), as indicated by a significant main effect of time (*F*_5,68_ = 20.8, *P* < 0.001), but not of group (*F*_1,14_ = 0.7, *P* = 0.4), and no significant time × group interaction (*F*_5,68_ = 0.8, *P* = 0.6). Thus, blockade of 5-HT_1A_ receptors facilitates LTP, but 5-HT_1A_ receptor activation does not result in further inhibition of LTP, similar to the effects noted above with direct 5-HT application in V1.

### 3.5. Effect of 5-HT_1A_ Blockade on LTP in Juvenile Rats

**Figure 7:**
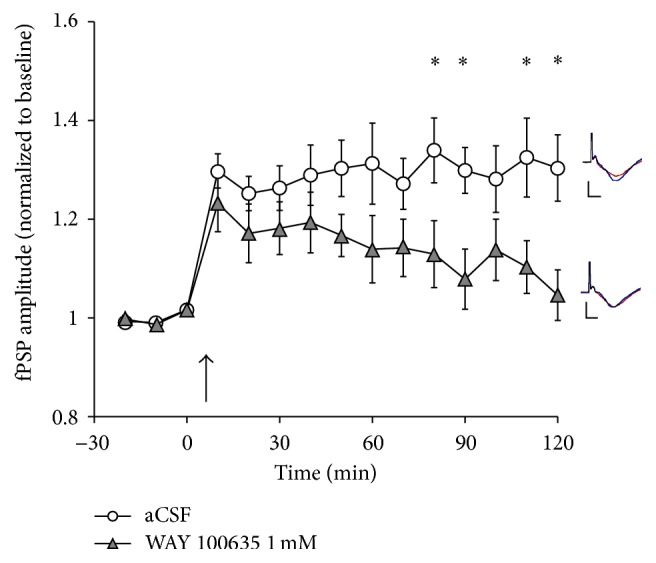
The effect of application (in V1 by means of reverse microdialysis) of either aCSF (*n* = 7) or WAY 100635 (1 mM, *n* = 8) on LTP following TBS (at arrow) of the LGN in juvenile (mean age 45 days, range 42–48 days), urethane-anesthetized rats. Application of WAY 100635 resulted in a suppression of LTP relative to animals receiving aCSF during four of the last five 10 min recording epochs relative to animals receiving aCSF. Inserts depict typical fPSPs before (red) and after (blue) LTP induction in the presence of aCSF (top) and WAY 100635 (bottom; calibration bars indicate 0.5 mV vertical and 10 ms horizontal; each fPSP trace is an average of 30 min of continuous recording). ^*∗*^ indicates a significant (*P* < 0.05, *t*-test) difference between the two groups.

Considerable evidence from* in vitro *experiments suggest that 5-HT plays a role in developmental plasticity, including the timing and closure of the “critical period” for plasticity in the rodent V1 [8, 9, 12, 17, 23, 27, 29, 31]. Thus, we also investigated the effects of 5-HT_1A_ receptor blockade by WAY 100635 application (1 mM) in a group of juvenile animals (age range: 42–48 days). In juvenile control animals (*n* = 7; mean age 44 days) receiving aCSF application in V1, TBS resulted in LTP, with fPSP amplitude at 130 ± 7% of the baseline during the final 30 min of the experiment (Figure 7). Surprisingly, in the presence of WAY 100635, juvenile rats (*n* = 8; mean age 44.9 days) showed reduced LTP, with fPSP amplitude at 110 ± 6% of baseline during the last 30 min of the experiment (Figure 7). Levels of LTP in these two groups of juvenile rats were different, as highlighted by a main effect of time (*F*_4,47_ = 14.4, *P* < 0.001) and a significant time × group interaction (*F*_4,47_ = 2.984, *P* < 0.03), even though the main effect of group only approached statistical significance (*F*_1,13_ = 4.3, *P* = 0.058). Together, these observations suggest that there is a developmental switch in the role of 5-HT_1A_ receptors in regulating V1 plasticity, with 5-HT_1A_ receptor activation promoting plasticity in the juvenile V1, while assuming an inhibitory role in V1 of adult, fully matured animals.

### 3.6. Effects of 5-HT and 5-HT Antagonists on the ECoG in V1

**Figure 8:**
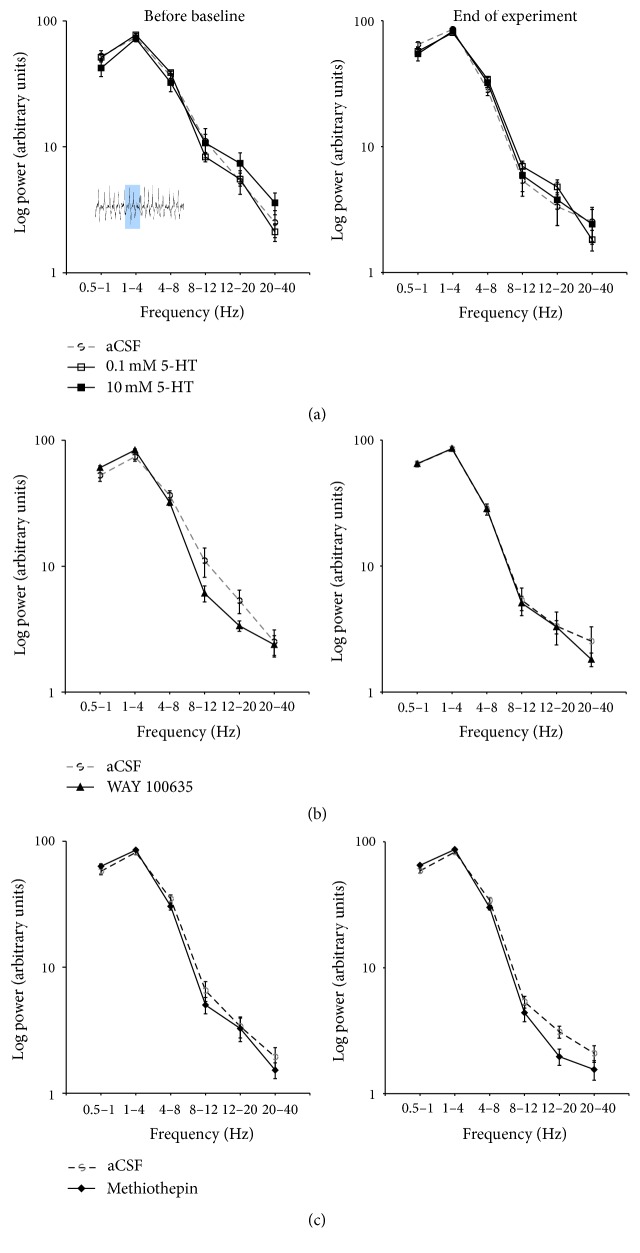
The effect of application (in V1 by means of reverse microdialysis; starting 20 min prior to the onset of recordings) of either aCSF, 5-HT (top; 0.1 and 10 mM), WAY 100635 (middle; 1 mM), or methiothepin (bottom; 1 mM) on electrocorticographic (ECoG) activity in V1 of adult, urethane-anesthetized rats. The ECoG was recorded and analyzed (5 sec epochs) by power spectral analysis before the onset of baseline recordings and at the end of the experiment. Throughout the experiment, ECoG activity was dominated by large amplitude, slow activity, with most power concentrated in the low delta (0.5–1 Hz) and delta (1–4 Hz) frequency bands. None of the pharmacological treatments resulted in a significant change in ECoG activity. Insert depicts typical ECoG activity in V1, with the shaded area representing a 5 sec epoch used for the analysis (group sizes the same as those in previous figures).

In addition to sampling evoked fPSPs, we also recorded ECoG activity in V1 to assess potential effect of 5-HT or drug application on spontaneous, oscillatory activity of the cortex. For all experiments, ECoG activity was assessed before the onset of baseline fPSP recordings (i.e., 20 min after the onset of drug application) and at the end of the experiment. In adult animals, the ECoG was dominated by large amplitude, slow oscillations, with peak power concentrated in the low delta (0.5–1 Hz) and delta (1–4 Hz) frequency bands, as determined by power spectral analyses (Figure 8). Power in all frequency bands (0.5–1 Hz, 1–4 Hz, 4–8 Hz, 8–12 Hz, 12–20 Hz, and 20–40 Hz) remained quite stable over the course of the experiment when aCSF was applied to V1 (Figure 8). Further, application of 5-HT, methiothepin, or WAY 100635 did not result in any significant changes in ECoG activity over the course of the experiment when compared with aCSF application (Figure 8; all group effects and group by time effects nonsignificant, *P*'s > 0.05).

Similar observations were made for ECoG activity in juvenile rats, which also exhibited peak power in the low delta (0.5–1 Hz) and delta (1–4 Hz) frequency bands that was not significantly altered by application of WAY 100635 (data not shown). These data suggest that changes in spontaneous, oscillatory activity in V1 do not account for the effects of methiothepin and WAY 100635 to alter LTP in V1 following TBS of the LGN in urethane-anesthetized animals.

## 4. Discussion

The present set of experiments examined the role of 5-HT and 5-HT_1A_ receptors in gating thalamocortical plasticity between LGN and V1 of juvenile and adult rats studied under urethane anesthesia. Application of 5-HT (0.1 to 10 mM) in V1 did not affect the induction or maintenance of LTP in adult animals under the present, experimental conditions. However, V1 application of the broad-acting 5-HT receptor antagonist methiothepin or the selective 5-HT_1A_ receptor antagonist WAY 100635 resulted in a clear facilitation of LTP in adult rats, suggestive of a suppression of LTP by endogenous 5-HT release and 5-HT receptor activation. In contrast, WAY 100635 reduced LTP when tested in a group of juvenile rats (mean age 45 days). None of the pharmacological treatments that altered LTP induction exerted significant effects on baseline synaptic transmission (fPSP amplitude prior to LTP induction; note that 5-HT at 10 mM suppressed baseline fPSP amplitude but did not affect LTP). Similarly, none of the pharmacological treatments altered spontaneous ECoG activity recorded in V1 over the course of the experiment. Together, these results indicate that 5-HT_1A_ receptors play an important, age-dependent role in gating plasticity in the thalamocortical visual system of rats, with 5-HT_1A_ receptor activation facilitating LTP in juveniles, but inhibiting LTP in the brains of adult animals.

We were surprised that, in our experiments, 5-HT application exerted no detectable effect on LTP induction in V1. A considerable amount of* in vitro *work has shown that 5-HT can alter LTP in V1 slice preparations, even though there are considerable inconsistencies in the specific results among various studies. In V1 slices of kittens, 5-HT has been shown to enhance LTP recorded in layer 4, effects that were absent in slices obtained from adult animals [8, 9]. Depletion of 5-HT (in combination with noradrenaline depletion) has been shown to impair LTP in layers 2/3 of the immature rat V1* in vitro, *an effect that was mimicked by application of either 5-HT_1A_ or 5-HT_2_ receptor antagonists [29]. Recently, these findings have been extended by showing that bath application of 5-HT enhances (in fact, reinstates) LTP in layers 2/3 of V1 of adult (8–10 weeks old) rats, confirming that 5-HT can exert a facilitating effect on the induction of long-lasting plasticity in V1 [12].

In clear contrast, other studies have indicated that 5-HT application results in a pronounced suppression of LTP at synapses between layers 4 and 2/3 of V1 of rats (3–5 weeks old), an effect that is mediated by both 5-HT_1A_ and 5-HT_2_ receptors [10, 30, 40, 41]. In fact, in V1 slices of 5-week-old rats, LTP could no longer be induced; however, acute bath application of the 5-HT receptor antagonist methysergide, acute 5-HT depletion, or neurotoxic ablation of serotonergic neurons restored LTP [10, 31]. A similar, inhibitory effect of 5-HT application has also been demonstrated for the induction of long-term depression in V1* in vitro *[42]. Interestingly, 5-HT levels in V1 show a significant increase (up to 3.5-fold from 3 to 5 weeks of age) over postnatal development [10, 31]. These observations have led to the hypothesis that 5-HT, in concert with other neurochemical changes (particularly the maturation of GABAergic circuits [43, 44]), contributes to the loss of V1 plasticity over postnatal life, resulting in the closure of the “critical or sensitive” period of V1 development [10, 31, 42].

Our data are consistent with the hypothesis that the release of endogenous 5-HT exerts a tonic, inhibitory influence on LTP induction, at least in adult rats. Cortical 5-HT is detectable in urethane-anesthetized rats, even during periods of ECoG synchronization [45]. We speculate that, under the present conditions, this effect of endogenous 5-HT is maximal, making the application of exogenous 5-HT ineffective in further suppressing LTP. However, future studies using 5-HT-depleted animals are necessary to examine this hypothesis. The presumed, inhibitory effect of endogenous 5-HT is relieved by methiothepin and WAY 100635, indicative of a role of 5-HT_1A_ receptors, even though we do not rule out the involvement of other 5-HT receptor types (e.g., 5-HT_7_ receptors, which are blocked by methiothepin and have recently been implicated in processes of learning, memory, and synaptic plasticity [35]). In V1 slices of young (5 weeks old) rats, both 5-HT_1A_ and 5-HT_2_ receptors mediate the effect of 5-HT to suppress LTP induction [10, 40, 41]. It is noteworthy, however, that WAY 100635 and methiothepin application resulted in very similar levels of LTP enhancement, indicating that 5-HT_1A_ receptor blockade alone is sufficient to result in a substantial disinhibition of LTP induction mechanisms under the present, experimental conditions.

In contrast to the enhancement of LTP in adult animals, application of WAY 100635 in juvenile animals (42–48 days) suppressed LTP, indicative of an age-related switch in the role of 5-HT_1A_ receptors in gating V1 plasticity. A similar phenomenon has previously been described for direct 5-HT application, albeit in a direction opposite to that revealed by the current set of experiments. Park et al. [12] observed that, in V1 slices obtained from juvenile rats (5 week old), 5-HT suppressed LTP, while 5-HT enhanced LTP in slices of adult animals (8–10 weeks old). At present, it is not clear why this pattern contradicts the data obtained in our experiments. However, it is noteworthy that the effects noted by Park et al. were mediated by 5-HT_2_ receptors [12], while out data clearly indicate a role for 5-HT_1A_ binding sites in V1 (see above). Thus, it is possible that different receptors populations do, indeed, exert opposing effects on plasticity gating in V1, an assumption that requires a critical assessment with further investigations (also see [46] for a differential effect of 5-HT_1A_ and 5-HT_2_ receptor activation on inhibitory transmission in V1).

To the best of our knowledge, the current experiments are the first to assess the role of 5-HT in gating LTP in the rodent V1 under* in vivo *conditions. Work conducted* in vitro* clearly offers significant advantages in terms of delineating microscopic and mechanistic properties of synaptic transmission and plasticity, such as the concurrent analysis of changes in inhibitory and excitatory synapses in V1 during LTP induction [47]. At the same time, there appear to be some important differences between LTP studied* in vivo *compared to* in vitro *conditions. Numerous studies have shown that V1 synapses studied* in vitro* become increasingly resistant to LTP induction with postnatal maturation, and slices harvested from adult animals do not show LTP with standard induction protocols [7, 8, 10–12, 31, 48, 49]. In sharp contrast, LTP is readily induced and maintained in V1 when studied in adult, intact-animal (anesthetized) preparations [13–16, 18, 21]. Also of interest, protocols that effectively elicit LTD in V1* in vitro* often fail to do so when tested under* in vivo *conditions [50, 51]. Thus, there clearly are some fundamental differences in the mechanisms and/or conditions that govern the induction of long-lasting plasticity at V1 synapses between* in vitro *and* in vivo *conditions, which might explain some of the apparent discrepancies among studies (see above). The use of tissue harvested from very young animals (20 days old or less), the routine use of some pharmacological agents in the bath solution (e.g., GABAergic antagonists), the removal of long-range (corticocortical, thalamocortical, and subcortical) projections, and the loss neuromodulatory inputs in slice preparations all introduce conditions that are very different from those present* in vivo *(see [52] for a detailed discussion of the advantages and disadvantages of electrophysiological work conducted* in vivo *and* in vitro*). These questions and discrepancies highlight the need for further work, in particular in intact animals, which preserve the anatomical connectivity and complex, physiological interactions among cortical and subcortical networks [52] that regulate activity and plasticity of synapses in V1 and elsewhere. Importantly, anesthetized and nonanesthetized preparations should also be compared to assess whether the systemic effects of urethane or other anesthetics alter the plasticity properties of V1 synapses [51, 52].

The results of the present experiments indicate that 5-HT_1A_ receptors exert a tonic, inhibitory influence over LTP in adult animals but facilitate LTP in the juvenile V1. Prior work has shown that 5-HT_1A_ receptors are located (albeit in different densities) on both interneurons and pyramidal cells of V1 [53]. Thus, direct effects on principal neurons and/or the modulation of inhibitory tone in V1 are likely candidate mechanisms for the effects noted in our experiments. The observation that 5-HT_1A_ receptor activation can suppress NMDA receptor functions in principle V1 cells [54] suggests a relatively direct action, but this does not preclude an involvement of other mechanisms (e.g., disinhibition or changes in the excitatory-inhibitory balance [47, 53]) in the effects observed here.

For the present experiments, we employed drug concentrations that are higher than those used for typical* in vitro *experiments. There are several reasons why we decided to use these higher concentrations: (a) pharmacological agents were applied by means of reverse microdialysis; it is generally assumed that only about 10% of drug molecules will diffuse across the dialysis probe membrane and into the surrounding, neural tissue [38, 39]; (b) drugs applied under* in vivo *conditions by reverse dialysis (but also during direct infusions) undergo extensive degradation, due to interactions with the probe membrane and lipophilic molecules, as well as diffusion and continuous enzymatic breakdown [38, 55]. Thus, as has been pointed out by others [55, 56], there is a clear discrepancy in terms of effective drug concentrations when comparing experimental* in vivo* and* in vitro* approaches. Nevertheless, we do acknowledge that it will be important for future work to establish whether the effects reported here can be elicited with drug concentrations that are lower than those employed for the present set of experiments.

There is a growing body of evidence that 5-HT plays an important role in shaping plasticity of the developing and mature nervous system and that alterations in 5-HT transmission can result in neurodevelopmental and psychiatry disorders [57, 58]. Prior work has shown that LTP in V1, in addition to its important role in ocular dominance plasticity [6, 11, 59], may also mediate processes of visual (perceptual, recognition) learning and memory storage [59, 60]. Based on these hypotheses and the results of the present investigation, we anticipate that altering serotonergic transmission in V1 exerts profound, age-dependent effects on visual processing and learning. For example, blockade of 5-HT_1A_ receptors in V1 may enhance perceptual learning in adults, when 5-HT acts to stabilize synaptic connectivity; opposite behavioral effects would be expected in juvenile animals, when 5-HT_1A_ receptors act to facilitate plasticity induction in V1. Clearly, investigations that involve a combination of behavioral, pharmacological, and electrophysiological approaches are required to characterize the role of 5-HT in visual behavior and directly test some of the hypothesis stated above.

Finally, the modulatory effects of 5-HT on activity- and experience-dependent plasticity also are of relevance to the potential treatment of various nervous system disorders. For example, chronic treatment with a selective serotonin reuptake inhibitor has been shown to reinstate ocular dominance plasticity (and LTP) in the V1 and allow recovery from amblyopic visual deficits in adult rodents [11], highlighting the therapeutic potential of serotonergic manipulations that alter the plasticity potential of cortical circuits (but see [61]). It remains to be established whether the effects following chronic, serotonergic manipulations relate to the role of 5-HT in acute plasticity gating described with the present set of experiments.

## Abbreviations

Ach: Acetylcholine
aCSF: Artificial cerebrospinal fluid
ANOVA: Analysis of variance
ECoG: Electrocorticogram
fPSP: Field postsynaptic potential
i.p.: Intraperitoneal
LGN: Lateral geniculate nucleus
LTD: Long-term depression
LTP: Long-term potentiation
SEM: Standard error of the mean
SSRI: Selective serotonin reuptake inhibitor
TBS: Theta burst stimulation
V1: Primary visual cortex
5-HT: 5-Hydroxytryptamine.
